# Development of a highly specific serodiagnostic ELISA for West Nile virus infection using subviral particles

**DOI:** 10.1038/s41598-021-88777-5

**Published:** 2021-04-28

**Authors:** Keisuke Maezono, Shintaro Kobayashi, Koshiro Tabata, Kentaro Yoshii, Hiroaki Kariwa

**Affiliations:** 1 Laboratory of Public Health, Faculty of Veterinary Medicine, Hokkaido University, N18, W9, Kita-ku, Sapporo, 060-0818 Japan; 2 2Division of Molecular Pathobiology, Research Center for Zoonosis Control, Hokkaido University, N20, W10, Kita-ku, Sapporo, 001-0020 Japan; 3 National Research Center for the Control and Prevention of Infectious Diseases, Nagasaki University, 1-12-4 Sakamoto, Nagasaki City, 852-8523 Japan

**Keywords:** Viral epidemiology, West nile virus

## Abstract

West Nile virus (WNV), a member of the Japanese encephalitis virus (JEV) serocomplex group, causes lethal encephalitis in humans and horses. Because serodiagnosis of WNV and JEV is hampered by cross-reactivity, the development of a simple, secure, and WNV-specific serodiagnostic system is required. The coexpression of prM protein and E protein leads to the secretion of subviral particles (SPs). Deletion of the C-terminal region of E protein is reported to affect the production of SPs by some flaviviruses. However, the influence of such a deletion on the properties and antigenicity of WNV E protein is unclear. We analyzed the properties of full-length E protein and E proteins lacking the C-terminal region as novel serodiagnostics for WNV infection. Deletion of the C-terminal region of E protein suppressed the formation of SPs but did not affect the production of E protein. The sensitivity of an enzyme-linked immunosorbent assay (ELISA) using the full-length E protein was higher than that using the truncated E proteins. Furthermore, in the ELISA using full-length E protein, there was little cross-reactivity with anti-JEV antibodies, and the sensitivity was similar to that of the neutralization test.

## Introduction

West Nile virus (WNV) belongs to the genus *Flavivirus* in Family *Flaviviridae* and is a member of the Japanese encephalitis serocomplex group, which includes Japanese encephalitis virus (JEV), Murray Valley encephalitis virus, and Saint Louis encephalitis virus^1^. In nature, WNV is maintained in a cycle between vector mosquitos and reservoir birds and can cause lethal encephalitis in mammals, including humans and horses^2^. Before the early 1990s, WNV was limited to Africa, the Middle East, West Asia, and Europe^3^. In 1999, WNV was reported for the first time in New York City and spread rapidly across North and South America^3,4^. In Russia, since a human outbreak of WNV infection was reported in the Volgograd Region in 1999, the endemic area has spread nationwide, including the Far Eastern region^5–7^. Because of its wide distribution, a system that enables the detection of WNV in birds and mammals is required to facilitate epidemiological investigations^3,8^.

JEV, which also causes encephalitis, is currently endemic in East Asia^9^. Because WNV and JEV frequently exhibit cross-reactivity in serodiagnosis, the distinction of these viruses is important in areas endemic for WNV or JEV^1,10^. There are several serodiagnostic tests for WNV infections, such as the enzyme-linked immunosorbent assay (ELISA), neutralization test (NT), immunofluorescence assay (IFA), and hemagglutination-inhibition test. The NT is the most reliable and specific method for the serodiagnosis of flavivirus infection^11^. However, the NT is time-consuming and requires a Biosafety Level 3 facility to handle live WNV^11^. By contrast, ELISA is simple and safe, but it is hampered by cross-reactivity among flaviviruses^12,13^. Therefore, development of a simple and secure detection system that can distinguish between WNV and JEV is necessary for the serodiagnosis of WNV infection.

The virions of WNV are approximately 50 nm in diameter and contain a single-strand positive-sense RNA genome^14^. The viral genome is packaged with multiple copies of capsid protein within a host-derived lipid bilayer, surrounded by both envelope (E) and membrane (prM/M) proteins^15,16^. E proteins are located at the surfaces of virions as homodimers in parallel and mediate binding to the host receptors and viral membrane fusion during entry^15,17^. The E protein is also the major antigenic site for neutralizing antibodies^18^. The E protein consists of three ectodomains (Domain I–III) and a C-terminal region including two α-helical regions (H1 and H2) in the stem and transmembrane anchor regions (TM1 and TM2)^19^. Because the ectodomains have the major antigenic epitopes, E proteins are often used as antigens for serodiagnostics and vaccines^20–22^. The stem-anchor region is involved in the membrane incorporation of E protein, stabilization of prM-E interactions, and low-pH-induced membrane fusion associated with conformational changes^23–26^. The coexpression of prM and full-length E proteins in mammalian cells results in the production of subviral particles (SPs), which are composed of a viral envelope without capsid protein or viral RNA^27,28^. Because the antigenicity of SPs is similar to that of authentic virions, SPs are useful antigens for serological diagnosis^11,28–30^. As the epitope cross-reactive among flaviviruses is less exposed on SPs, an ELISA using SPs enabled the specific serodiagnosis of tick-borne encephalitis virus (TBEV) infection^11^. By contrast, coexpression of truncated E proteins lacking part or all of the C-terminal region and the prM protein suppressed the formation of SPs^24^. Truncation of the C-terminal region promoted more efficient secretion of the TBEV E protein compared to the full-length E protein^27^. However, little is known about the influence of truncation on the secretion and antigenicity of the E protein of WNV.

In this study, we constructed an expression system for full-length E protein and various truncated E proteins lacking the C-terminal regions with One-STrEP-Flag (OSF) tags and analyzed their properties and antigenicity. As a result, we developed a novel serodiagnostic system with high sensitivity and specificity comparable to those of the NT.

## Results

### Effects of deleting the stem-anchor region on the properties of E protein

**Figure 1:**
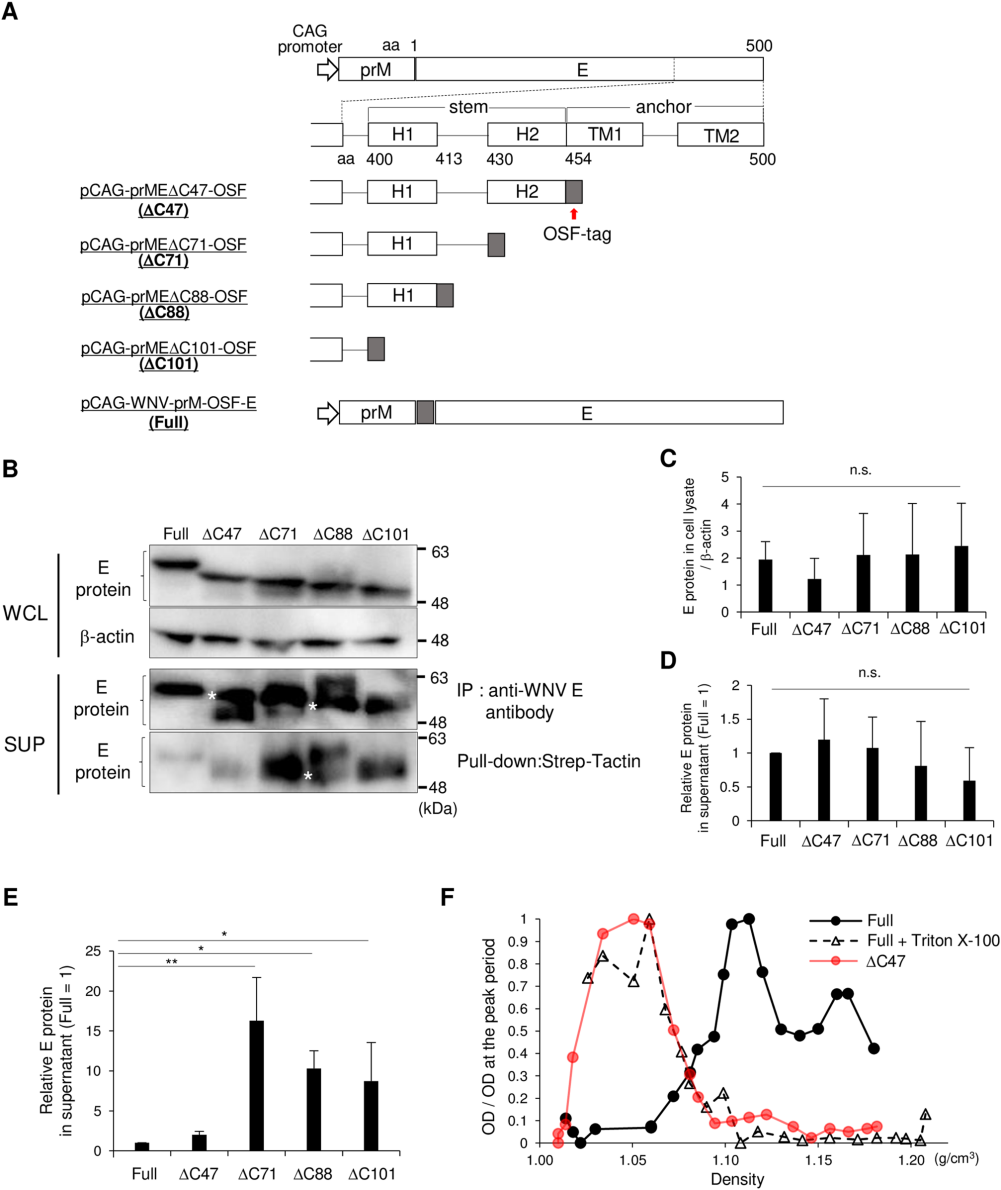
Properties of truncated and full-length E protein. (**A**) Schematic of plasmids expressing truncated or full-length E protein with an OSF tag. The OSF-tag sequence was fused to the C terminus of the truncated E protein or the N terminus of the full-length E protein. The amino-acid (aa) positions are shown. The E proteins expressed upon transfection with the plasmids are labeled “∆C47” for pCAG-prME∆C47-OSF, “∆C71” for pCAG-prME∆C71-OSF, “∆C88” for pCAG-prME∆C88-OSF, “∆C101” for pCAG-prME∆C101-OSF, and “Full” for pCAG-WNV-prM-OSF-E. (**B**–**G**) 293 T cells were transfected with plasmids expressing Full, ∆C47, ∆C71, ∆C88, and ∆C101 E proteins. (**B**) The E proteins in cell lysates (upper panel) and supernatants (lower panel) after precipitation using anti-WNV E antibody or Strep-Tactin were detected via Western blotting using anti-WNV E antibody. The white asterisk indicates a band of estimated molecular weight. Cropped blots are shown; full-length blots are presented in Supplementary Fig. S1A,B. (**C**) The relative level of each E protein is shown, as calculated by dividing the intensity of E protein in the cell lysate by that of β-actin. (**D,E**) Band intensities of the E proteins in the lower panel of (**B**) (immunoprecipitation by anti-WNV antibody and pull-down by Strep-Tactin) were measured, and relative intensities were calculated by normalization to the Full value. Data are presented as the mean ± standard error of four independent experiments. Statistical significance was evaluated using one-way ANOVA followed by the Dunnett test (*p < 0.05, **p < 0.01). (**F**) Supernatants from cells transfected with plasmids expressing full-length E protein with (black dotted line) or without (black line) TritonX-100 treatment or expressing ∆C47 (red line) were subjected to 10–50% sucrose density gradient ultracentrifugation, and the resulting fractions were examined for the presence of E protein by ELISA.

The C-terminal region of E protein consists of α-helical regions (H1 and H2) and transmembrane regions (TM1 and TM2) termed the stem-anchor region, which connects the E protein ectodomain to the viral membrane^24,27^. Deletion of the stem-anchor region or insertion of a sequence into the stem-anchor region affected the formation of viral particles, but the E protein was released extracellularly^27,31^. To examine the importance of the C-terminal region for the expression and release of E protein, we constructed plasmids expressing prM and various truncated E proteins with an OSF tag at the C terminus to retain the structure of the ectodomain. To expose the OSF tag on the surface of each particle, the sequence was inserted at the N terminus of full-length E protein as reported previously^11,31^ (Fig. 1A). Each plasmid was transfected into 293 T cells, and the amount of E protein in the cells or supernatant was determined. The expression level of each E protein in transfected cells was similar (Fig. 1B,C). After precipitation of E protein using an anti-WNV E antibody, the extracellular level of each E protein truncated at the C terminus was comparable to that of full-length E protein (Fig. 1B,D). By contrast, precipitation of E protein with Strep-Tactin showed that the extracellular levels of full-length E protein and ∆C47, lacking transmembrane regions, were less than those of the other truncated E proteins (Fig. 1B,E). These results suggest that the deletion of the C-terminal region did not affect the production of E protein, but it did affect protein reactivity with Strep-Tactin.

To assess the physical properties of the full-length and truncated E proteins, supernatants containing full-length E protein or ∆C47, lacking H2 and transmembrane regions, were sedimented via sucrose gradient, and the presence of E protein in the resulting fractions was examined by ELISA. The full-length E protein was mainly detected at a density of 1.12–1.16 g/cm^3^, which coincides with that of flavivirus SPs, and the E protein signal was shifted to low-density fractions following treatment with Triton X-100^11,32^ (Fig. 1F). ∆C47 was detected in the low gradient fractions similar to Triton X-100 treated full-length E protein (Fig. 1F). These results indicated that the full-length E protein was released into the supernatant as SPs, whereas E protein lacking a C-terminal region was unable to form SPs.

### Development of ELISA for serodiagnosis

**Figure 2:**
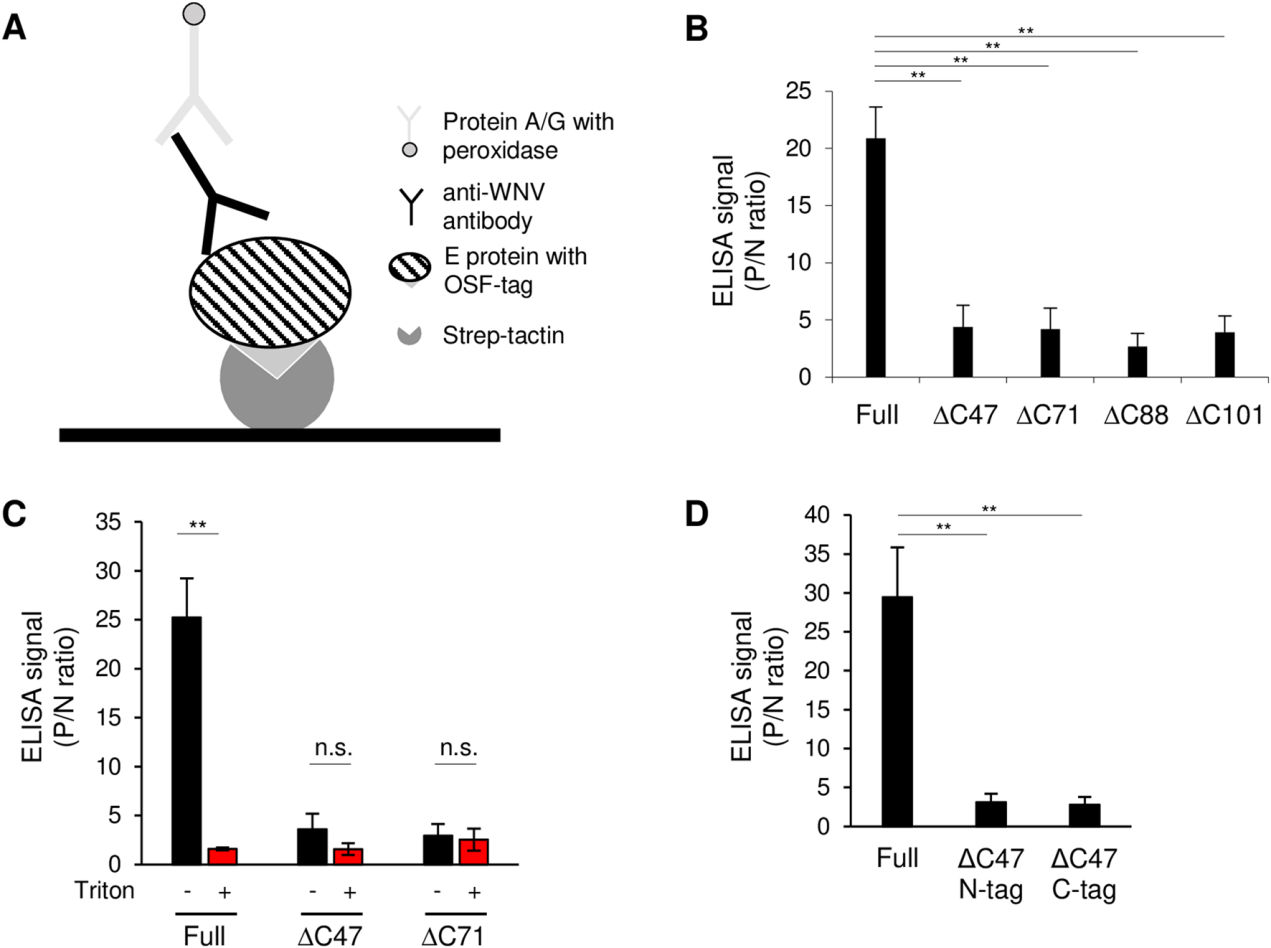
Development of an ELISA for the serodiagnosis of WNV infection. (**A**) Schematic of the developed ELISA using E proteins with an OSF tag. The E protein with an OSF tag is trapped by a Strep-Tactin coating. Anti-WNV IgG in WNV-infected mouse serum reacts with the E protein and is detected by protein A/G conjugated with HRP. (**B**) Comparison of E proteins as antigens for ELISA. The results were recorded as P/N ratio (OD value of E protein relative to that of the negative control antigen). Data are presented as the mean ± standard error of three independent experiments. Statistical significance was evaluated using one-way ANOVA followed by the Tukey–Kramer test. (**p < 0.01). (**C**) Comparison of reactivity with and without Triton X-100. The supernatant from transfected 293 T cells was treated with (red) or without (black) 1% Triton X-100 and used as the antigen in an ELISA. The results were recorded as P/N ratio. Data are presented as the mean ± standard error of three independent experiments. Statistical significance was evaluated using a two-tailed Student's t-test (**p < 0.01). (**D**) The ELISA signal using full-length (Full), ∆C47 with OSF tag at the N-terminus (∆C47 N-tag) or C-terminus (∆C47 C-tag) of E protein. The results were recorded as P/N ratio (OD value of E protein relative to that of the negative control antigen). Data are presented as the mean ± standard error of three independent experiments. Statistical significance was evaluated using one-way ANOVA followed by the Scheffe’s *F* test (**p < 0.01).

We previously reported that the Strep-SP ELISA using recombinant SPs with Strep-tag as the antigen enabled the detection of a TBEV-specific antibody^11^. To develop a novel ELISA for the serodiagnosis of WNV infection, E proteins were used as antigens for detecting an anti-WNV antibody. In this ELISA, each E protein with an OSF tag was captured by Strep-Tactin coated on a plate, and the antibody-E protein complex was detected using Protein A/G (Fig. 2A). The ELISA signal using full-length E protein was significantly higher than that using truncated E proteins (Fig. 2B), indicating that full-length E protein is suitable for detecting anti-WNV antibody.

To confirm the importance of particle formation by E proteins for the sensitivity of the ELISA, E proteins were treated with Triton X-100 and used for ELISA. The treatment significantly decreased the ELISA signal of full-length E protein, whereas the decrease in ELISA signal was not observed in ∆C47 or ∆C71 (Fig. 2C). These results suggest that the formation of SPs is important for the sensitivity of the ELISA. Therefore, we used full-length E protein with an OSF tag as an antigen for ELISA in subsequent experiments.

To confirm that the ∆C47 lacks ability of the formation of SPs and that effect of an OSF tag location on the ELISA, we performed the ELISA using ∆C47 inserted the OSF tag at the N-terminus of E protein (∆C47 N-tag). Consistent with the result of ∆C47 with the OSF tag at the C-terminus of E protein (∆C47 C-tag), the ELISA signal of ∆C47 N-tag was significantly lower than that of full-length E protein. This result suggested that the ∆C47 could not form SPs and that the location of the OSF tag unaffected the ELISA.

**Figure 3:**
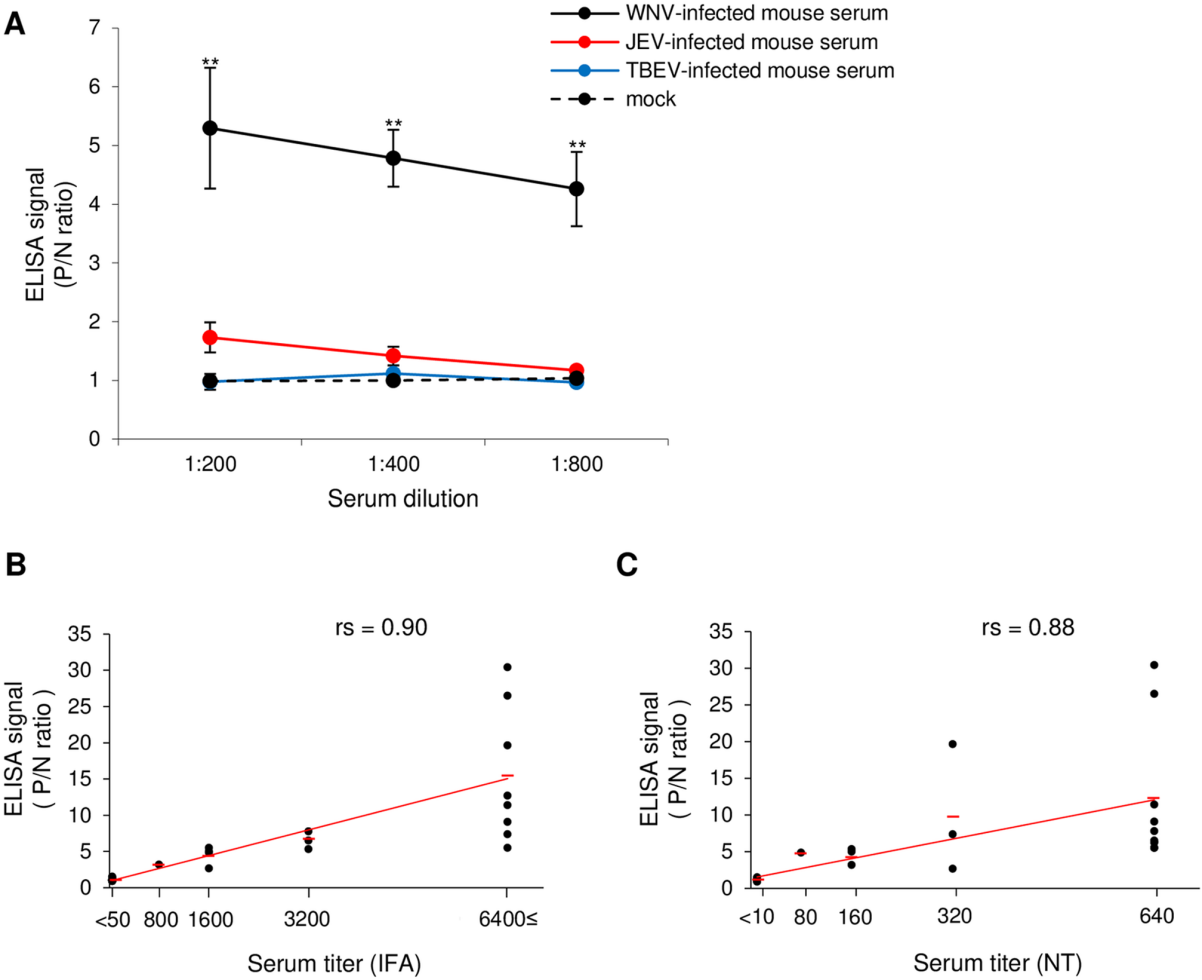
Sensitivity and specificity of the ELISA using full-length E protein. (**A**) Reactivity of WNV-infected (n = 3), JEV-infected (n = 3), and TBEV-infected (n = 3) mouse sera in the ELISA. Negative sera (mock; n = 3) were used as controls. Standardized serum from a WNV-, JEV-, or TBEV-infected mouse was serially diluted as described. The results were recorded as P/N ratios. The difference between the WNV-infected mouse serum and JEV-infected, TBEV-infected mouse serum, or mock was significant (**p < 0.01) according to the Tukey–Kramer test. (**B,C**) Correlation between the ELISA signal (P/N ratio) and results from the IFA or NT. Serum samples from mice infected with WNV were tested using ELISA and the results were compared to the titers estimated from the IFA (**B**) and NT (**C**). The strengths of the correlations (rs) were calculated as Spearman’s rank correlation coefficients (A, rs = 0.90; B, rs = 0.88). Red bars indicate approximate curves.

In serodiagnosis, cross-reactions frequently occur among sera from animals infected with flavivirus, especially following WNV and JEV infection. To examine the specificity of the ELISA, sera from mice infected with WNV, JEV, or TBEV were tested. The neutralizing antibody titer of each serum from mice inoculated with WNV, JEV, or TBEV was examined by neutralizing test. Each serum sample for ELISA was standardized by diluting to the same neutralization titer. The ELISA signal of sera from mice inoculated with WNV was significantly higher than that from mice inoculated with JEV, TBEV, or mock (Fig. 3A). No differences were observed in the ELISA signal of sera from mice inoculated with JEV or TBEV, or mock (Fig. 3A). These results suggest that an ELISA using the full-length E protein with an OSF tag can be highly specific for the detection of WNV infection.

To examine the sensitivity of the ELISA, serum samples from mice infected with WNV were tested using an IFA or NT and the results were compared with those of the ELISA. The ELISA signal was strongly correlated with the serum titers from the IFA (Fig. 3B) and NT (Fig. 3C). These results suggest that our ELISA using full-length E protein could be used to detect anti-WNV antibody in a manner comparable to that of the IFA and NT.

## Discussion

We analyzed the properties of OSF-tagged full-length WNV E protein and various truncated WNV E proteins lacking the C-terminal region and used them in a serodiagnostic ELISA for WNV infection. The C-terminal region of E protein influences particle formation but not the expression and secretion of E protein. Also, the structure of E protein was important for the sensitivity and specificity of the ELISA.

The ELISA using full-length E protein, which formed SPs, exhibited higher reactivity than that using truncated E proteins. Furthermore, disruption of the structure of full-length E protein decreased the reactivity to anti-WNV antibody in the ELISA, suggesting that the structural arrangement of E protein influenced the sensitivity of the ELISA. Several antibodies in flavivirus-infected serum could bind to not only the epitopes of E protein at the intra-dimer but also the epitopes at the inter-dimer^33–36^. Solidification of viral particles directly onto ELISA plates impaired the antigenic structure of the native epitopes of E protein^37^. Because full-length E protein, which formed SPs, was captured by Strep-Tactin on the plate, our system conserved the native epitopes and detected antibodies in serum with high sensitivity. Furthermore, the amount of full-length E protein precipitated by Strep-Tactin was less than that of truncated E protein, whereas ELISA using the full-length E protein exhibited higher reactivity than that using truncated E protein. These results suggest that the reactivity of ELISA in a single SP is much higher than that of a single monomer or dimer of E protein. Therefore, the structure of E protein was more important than its quantity for the sensitivity of the ELISA.

Our ELISA using OSF-tagged SPs exhibited little cross-reactivity with anti-JEV antibodies in mouse serum. The cross-reaction are often observed among flaviviruses^1^. Many serological tests such as commercial or in-house IgG-ELISAs or IFAs exhibited cross-reactivity among members of the JE serocomplex^10,38,39^. The cross-reactive epitopes are thought to be inaccessible on infectious virions or SPs but may be exposed by conformational changes in E protein during these assays, such as during plate coating or fixation^37,40,41^. In our ELISA, the capture of SPs with an OSF tag by Strep-Tactin might have conserved the native structure of the viral particles and epitopes.

Deletion of the stem and anchor regions of WNV E protein did not affect the expression and secretion of E protein, despite incomplete SPs formation. Deletion of the stem-anchor region of TBEV E protein increased the secretion of E protein, whereas deletion of dengue virus (DENV) E protein did not influence expression or secretion^27,42^. The role of the stem-anchor region in the expression and secretion of E protein may differ among flaviviruses. The transmembrane regions of E protein were required for the production of SPs of TBEV and yellow fever virus^24,43^. Our sucrose gradient ultracentrifugation results indicated that WNV E protein lacking both transmembrane regions could not form SPs, suggesting that the transmembrane regions of the WNV E protein are important for particle assembly. Furthermore, the sedimentation of full-length E protein generated two distinct peaks in the SPs fraction. In a previous study examining TBEV, SPs of two distinct sizes were separated by sucrose gradient centrifugation, of which the smaller particles were mature SPs and the larger particles were the immature form of SPs^44^. SPs produced from the full-length E protein of WNV seem to be include both the mature and immature forms. Investigating the antigenicity of each type of SP may facilitate the development of a detection system.

In conclusion, we demonstrated that WNV SPs with an OSF tag can be used in a highly sensitive and specific serodiagnostic system for WNV infection. Given the common properties of the SPs of flaviviruses, the system constructed in this study can be applied to the serodiagnosis of other flaviviruses^27,45–47^. WNV is transmitted between avian hosts and mosquito vectors in nature^48^. Further improvement and application of our ELISA serodiagnostic system to detect avian infection by WNV will facilitate investigations of the epidemiology of WNV in nature.

## Methods

### Cells

Human embryonic kidney 293 T (293 T) cells were cultured at 37 ℃ in high-glucose Dulbecco’s modified Eagle’s medium (Wako, Osaka, Japan) supplemented with 10% fetal bovine serum (FBS) and penicillin/streptomycin. Vero cells and baby hamster kidney fibroblast (BHK cells) were cultured in Eagle’s minimum essential medium (Wako) containing 10% FBS and used for neutralization tests. Expi293F cells were cultured in Expi293 Expression medium (Thermo Fisher Scientific, Waltham, MA) at 37℃ with 8% CO_2_ and shaking at 125 rpm.

### Viruses

The WNV 6-LP strain was established from the WNV NY99-6922 strain as described previously^49^. The JEV JaGAr-01 strain or the TBEV Oshima 5–10 strain was propagated in BHK cells. All WNV and TBEV experiments were performed at a Biosafety Level-3 facility at Hokkaido University, Japan, according to the institutional guidelines, as described previously^50^.

### Serum

Eight-week-old female BALB/c mice were purchased from Japan SLC Inc. (Shizuoka, Japan). Mice were anesthetized using sevoflurane and inoculated subcutaneously in the back region with 100 plaque forming units (PFU)/0.1 mL of WNV (n = 3) or 10^5^ PFU/0.1 mL of JEV (n = 3), or 10^4^ PFU/0.1 mL of TBEV (n = 3). The mice were housed in a climatically controlled environment (24 ± 2 °C) under a 12 h (7 am/7 pm) light/dark cycle with access to chow and water ad libitum. The mice were euthanized when more than 10% of their body weight was lost, and blood samples were collected from the heart under anesthesia and kept at 4 ℃ overnight. The sera were collected and inactivated for 30 min in a heat block at 56 ℃ and stored at − 80 ℃ until use.

### Construction of plasmids

The pCAG-WNV-prME plasmid was constructed by subcloning the PCR-amplified prME coding regions of WNV. The sequence of the OSF tag was amplified using PCR from the pCAG-OSF plasmid, generously provided by Dr. Kamitani (Gunma University, Japan), and it was then introduced at the N terminus of the E protein in the pCAG-WNV-prME plasmid using an In-Fusion HD Cloning kit (TaKaRa Bio USA, Inc., Mountain View, CA) and named pCAG-WNV-prM-OSF-E.

Forty-seven amino acids were truncated from the C terminus of the E protein in pCXSN-prME, which was constructed previously^51^ using appropriate primers via inverse PCR and named pCXSN-prME∆C47. The OSF tag was inserted at the C terminus of the E protein in pCXSN-prME∆C47 using an In-Fusion HD Cloning kit (TaKaRa Bio USA, Inc.) and named pCXSN-prME∆C47-OSF. The sequence of prME∆C47-OSF was amplified using PCR from pCXSN-prME∆C47-OSF and cloned into the pCAGGS vector plasmid. The resultant plasmid was named pCAG-prME∆C47-OSF. The pCXSN-prME∆C71-OSF, pCXSN-prME∆C88-OSF, and pCXSN-prME∆C101-OSF plasmids were constructed from pCXSN-prME∆C47-OSF via inverse PCR using appropriate primers. The sequence of the insert of each plasmid was amplified using PCR and cloned into the pCAGGS vector plasmid; the resulting plasmids were named pCAG-prME∆C71-OSF, pCAG-prME∆C88-OSF, and pCAG-prME∆C101-OSF, respectively. The pCAG-prM-OSF-E∆C47 was constructed from pCAG-WNV-prM-OSF-E via inverse PCR using appropriate primers.

### Transfection

Plasmids were transfected into 293 T or Expi293F cells using X-tremeGENE HP DNA Transfection reagent (Roche, Basel, Switzerland) or an ExpiFectamin 293 Transfection kit (Thermo Fisher Scientific) according to the manufacturer’s instructions. Transfected 293 T and Expi293F cells and culture supernatant were harvested at 48 h post-transfection and stored at − 80℃.

### SDS-PAGE and immunoblotting

Transfected cells were washed and lysed with lysis buffer (1% Triton X-100, 50 mM Tris–HCl [pH 7.5], 1 mM ethylenediaminetetraacetic acid, and 0.25 M sucrose). The lysates were prepared as described previously^51^. The supernatant from transfected cells was precipitated as described below.

Samples were electrophoresed in polyacrylamide-sodium dodecyl sulfate gels and transferred to polyvinylidene difluoride membranes. Immunoblotting was performed as described previously^51^. Briefly, blocked membranes were incubated overnight with an anti-WNV E mouse antibody (1:1000; Millipore, Darmstadt, Germany). Complexes were detected using horseradish peroxidase (HRP)-conjugated anti-mouse IgG secondary antibody (1:10,000). Protein bands were visualized as described previously^51^.

### Protein precipitation

Culture supernatants of transfected 293 T cells were harvested and treated with 1% Triton X-100. The supernatants were immunoprecipitated or pulled down using an anti-WNV E antibody (Millipore) or Strep-Tactin Sepharose (IBA Lifesciences, Gottingen, Germany). Immunoprecipitation was performed by incubating the supernatants at 4 ℃ for 3 h with 20 μL of antibody-coupled Surebeads Protein G Magnetic Beads (Bio-Rad, Hercules, CA). The supernatants were next incubated with Strep-Tactin Sepharose at 4 ℃ for 2 h. The reaction and elution steps were performed following the manufacturer’s instructions. The precipitated protein was separated using SDS-PAGE and analyzed by immunoblotting as described above.

### Sucrose density gradient ultracentrifugation

Culture supernatants of transfected 293 T cells were treated with or without 1% Triton X-100 and were analyzed via ultracentrifugation in a 10–50% sucrose gradient in HEPES buffer (50 mM HEPES [pH 7.8] and 140 mM NaCl) at 110,000×*g*, at 4 °C for 16 h in a CP80MX centrifuge using the P40ST-1239 rotor (Hitachi Koki, Tokyo, Japan). Fractions of 550 μL were harvested from the top layer, and the amount of E protein in each fraction was measured by ELISA.

### Enzyme-linked immunosorbent assay

Ninety-six-well ELISA plates (Corning, New York) were coated with Strep-Tactin AP conjugate (1:1000; IBA Lifesciences), incubated overnight at 4 ℃, and blocked with Block Ace (DS Pharma Biomedical, Osaka, Japan). After washing with PBS containing 0.05% Tween 20, culture supernatants containing the constructed E proteins with an OSF tag as an antigen were added, followed by serum samples (1:200). The antibody for WNV, JEV, or TBEV was detected using Protein A/G conjugated to HRP (1:1000; Thermo Fisher Scientific), with OPD as the substrate. Negative control antigens were prepared from the supernatant of untransfected 293 T or Expi293F cells. The results were calculated as P/N ratios (comparing the optical density [OD] values of E proteins to that of the negative control antigen).

### Immunofluorescence assay

Vero cells were plated at a density of 1 × 10^5^ cells/well into Nunclon Delta 96-well plates (Thermo Fisher Scientific) and incubated overnight. Cells in the plates were infected with WNV and incubated at 37 ℃ for 2 days. Infected cells were fixed in 4% paraformaldehyde for 15 min and then washed with PBS. Fixed cells were permeabilized and blocked by incubating in PBS containing 0.1% Triton X-100 and 1% bovine serum albumin (BSA) for 30 min. The cells were incubated with WNV-infected serum diluted in PBS containing 0.1% Triton X-100 and 1% BSA for 1 h. After washing with PBS, cells were incubated with Alexa Fluor 555 goat Anti-Mouse IgG (H + L) Secondary Antibody (Life Technologies, CA). All procedures were performed at room temperature. Cells were observed using an IX71N-22FL/PH system (Olympus, Tokyo, Japan), and images were processed using Standard CellSens (Olympus).

### Neutralization assay

WNV, JEV, or TBEV particles were incubated with serially diluted sera and inoculated in Vero or BHK cells, respectively. The cells were incubated in a medium containing 1.25% methyl cellulose and 5% FBS for 4 days. Next, the cells were fixed with 10% formalin and stained with 0.1% crystal violet. Serum samples that exhibited an 80% reduction in plaque formation of WNV, JEV, or TBEV were screened. The neutralization titers of mouse sera infected with WNV, JEV, or TBEV were calculated (WNV: 1:320, JEV: 1:40, TBEV: 1:320), then each serum was standardized by dilution with PBS to the same neutralization titer.

### Quantification and statistical analysis

Band intensity was quantified via densitometric measurement of the lanes using Image Lab Software (Bio-Rad) and normalized to the relative density of the loading control in the same blot. The optical density at 450 nm was recorded using a Viento 808 microplate reader (DS Pharma Biomedical Co., Ltd, Osaka, Japan).

Data were compared among multiple independent groups using one-way ANOVA followed by the Tukey–Kramer or Dunnett test. Statistical significance was established a priori at p < 0.05.

### Ethics statement

Animal experiments were performed according to the basic guidelines for animal experiments of the Ministry of Education, Culture, Sports, Science and Technology of Japan. The experimental protocols were approved by the Animal Care and Use Committee of Hokkaido University (approval number: 19-0142) and complied Animal Research: Reporting of In Vivo Experiments (ARRIVE) guidelines.

## Supplementary Information

Supplementary Information.
