# Supplementary material for: Development of a highly specific serodiagnostic ELISA for West Nile virus infection using subviral particles
Source: Sci Rep. 2021 Apr 28;11:9213. doi: 10.1038/s41598-021-88777-5 (PMC8080695; doi:10.1038/s41598-021-88777-5)
Supplement: Supplementary file 1 — Supplementary Information. [file 41598_2021_88777_MOESM1_ESM.pdf]

Development of a highly specific serodiagnostic ELISA for West Nile virus infection using subviral particles

Keisuke Maezono[1], Shintaro Kobayashi[1*], Kentaro Yoshii[1], Koshiro Tabata[2], Hiroaki Kariwa[1]

1. Laboratory of Public Health, Faculty of Veterinary Medicine, Hokkaido University, N18, W9, Kita-ku, Sapporo, 060-0818, Japan
2. Division of Molecular Pathobiology, Research Center for Zoonosis Control, Hokkaido University, N20, W10, Kita-ku, Sapporo, 001-0020, Japan

Supplementary Figure S1

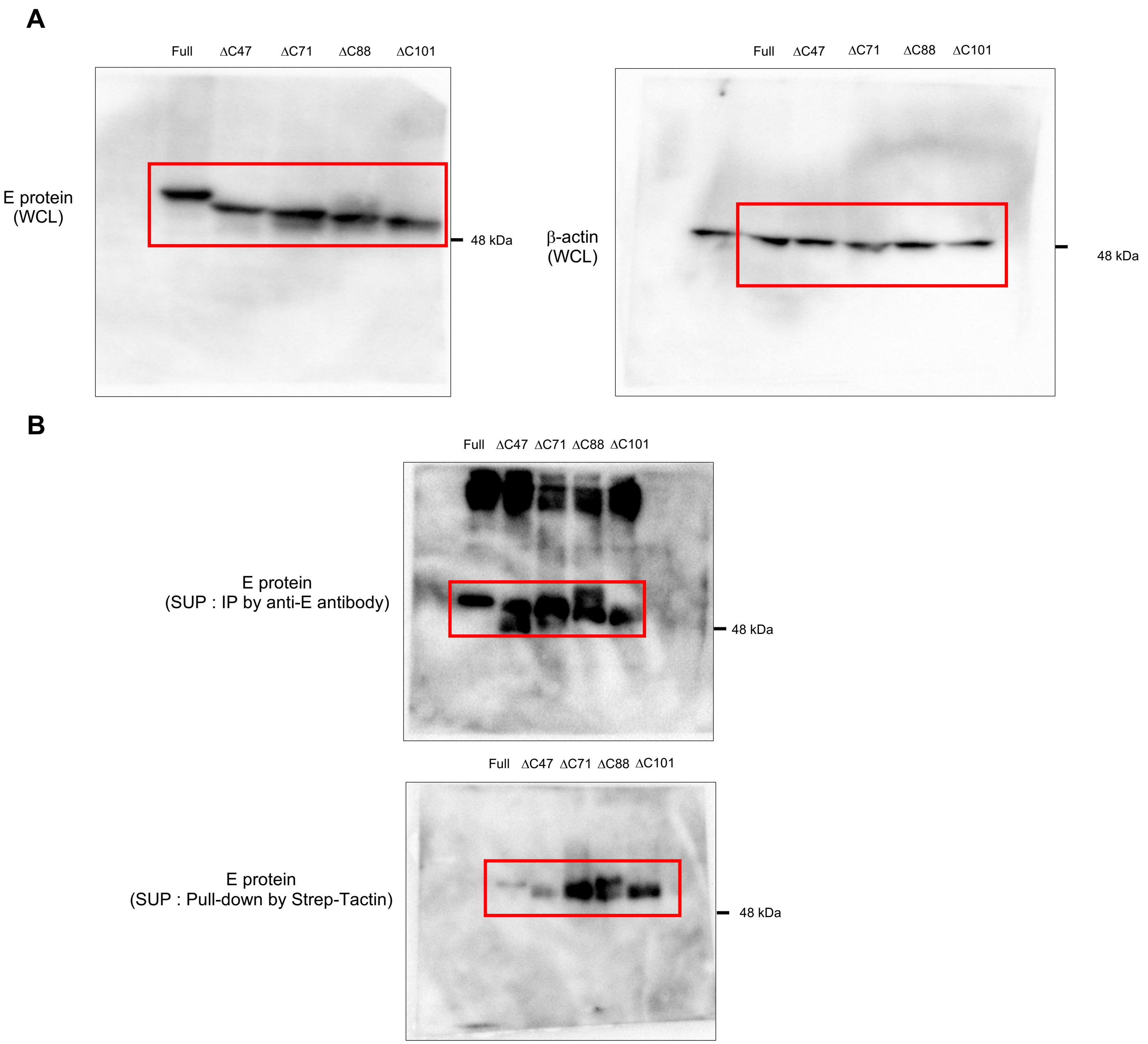

Fig. S1 Full-length blots of Figure. 1
The red-boxed regions were shown in Fig. 1B (Figs. S1A and S1B)
